# Informed consent: Navigating the bioethical complexities of clinical research

**DOI:** 10.6026/973206300212950

**Published:** 2025-09-30

**Authors:** Ravindra Waykar, Yogesh A. Kulkarni

**Affiliations:** 1 Caspersen School of Graduate Studies, Drew University, 36 Madison Ave, Madison, NJ 07940, United States; 2 Shobhaben Pratapbhai Patel School of Pharmacy & Technology Management, SVKM's Narsee Monjee Institute of Management Studies (NMIMS) Deemed to be University, Mumbai, India

**Keywords:** Informed consent, clinical trials, ethical framework, belmont report, nuremberg code, declaration of helsinki, research ethics

## Abstract

Informed consent (IC) has traditionally been regarded as the fundamental principle of ethical clinical research involving human subjects. This article explores the limitations of the IC, assesses the need for a comprehensive ethical framework and proposes a roadmap for developing a framework that holistically safeguards participants' rights, safety and well-being. The evolving nature of medical technology and complex decision-making situations necessitate a more comprehensive approach to research ethics. This review emphasizes the need for a procedural plan to structure moral deliberations and address potential conflicts between ethical values. By integrating ethical values and benchmarks, a systematic and consistent approach is required to uphold ethical integrity in clinical research beyond the limitations of informed consent.

## Background:

The ethical framework guiding the principal investigator's conduct has long been determined by the fundamental principles of ethics outlined in foundational documents such as the Belmont Report, the Nuremberg Code, the Declaration of Helsinki, the International Code of Medical Ethics and others that emphasize informed consent (IC), beneficence and justice [1, 2]. Although these guidelines have significantly influenced how health professionals' approach clinical trials and other research practices, over-reliance on IC, especially in studies involving human subjects, has proven problematic in addressing complex ethical challenges in modern clinical trials [3, 4]. Specifically, reliance on IC as the key ethical principle in clinical research is problematic due to the lack of standard criteria to certify what constitutes it, how informed consent forms (ICF) should be written and presented to potential participants and instances in which participants with inadequate decisional capacity consent to research [5, 6, 7-8]. Notably, such challenges create loopholes that can be exploited to undermine participants' rights, safety and well-being [9, 10, 11, 12-13]. Furthermore, evolving ethical standards and historical injustices to research participants have compounded mistrust among particular populations, complicating the ethical landscape of clinical research [14, 15, 16, 17-18]. While IC is essential, it does not suffice to guarantee ethical clinical research involving human participants; therefore, a systematic and consistent framework that integrates all pertinent ethical factors is essential to uphold the integrity of clinical trials in terms of safeguarding human subjects' rights, safety and well-being [19, 20, 21, 22-23]. Although extensive ethical guidelines and regulatory frameworks have been developed by regulatory authorities, they do not provide a holistic ethical approach from the medical, health and humanities perspectives, which is the foundation of ethical research [24, 25, 26, 27- 28]. While essential in clinical research, as a single ethical principle, it is not adequate to ensure ethical integrity in research involving humans [29, 30- 31]. Specifically, the IC's limitations are linked to its inconsistency in addressing issues such as fair and equitable subject selection, establishing systemic risk-benefit analysis beyond individual autonomy concerns and ensuring ethical compliance in ongoing clinical research [32, 33, 34-35]. This article explores the limitations of IC as the cornerstone of clinical research ethics, assess the need for a comprehensive ethical framework for clinical research and provide a roadmap for developing a comprehensive ethical framework that extends the IC to holistically safeguard subjects rights, safety and well-being in clinical studies. Therefore, it is of interest to have a single, consistent and reliable ethical framework that integrates all pertinent principles and benchmarks to provide a comprehensive ethical framework that can address all ethical challenges that continue to emerge in dynamic clinical settings.

## Historical context of ethical regulations in clinical trials:

The ethical foundations guiding clinical research in contemporary science have developed and continue to evolve since the 19th century [36, 37-38]. The Nuremberg Code is a pioneer in modern research ethics [39, 40- 41]. A historical normative analysis of the global development of research ethics regulations and review structures provides a detailed account of the evolution of research ethics oversight [42, 43, 44-45]. The creation of the Nuremberg Code and other regulatory systems was driven by a combination of medical controversies and the necessity to align with advancements in the United States [46, 47- 48]. McNair supports Israel's argument by affirming that the creation of the Nuremberg Code in 1947 was part of the reaction by American judges to the unethical conduct of Nazi doctors on Holocaust victims [49]. Subsequent attempt to improve ethics in medical research manifested in the development of the Declaration of Helsinki (1964) and the Belmont Report (1979) [50, 51-52]. A narrative review by McNair (2022) explains that the Declaration of Helsinki (1964) was developed by the World Medical Association (WMA) to improve areas not properly covered by the Nuremberg Code to minimize ethical risks amid growing medical research and ethical violations in the third and fourth quarters of the 20th century [49]. After the enactment of the National Research Act in 1974, following indignation over the Tuskegee Syphilis Study, the Belmont Report was developed in 1979 as part of the federal government's strategy to upgrade the ethical codes governing clinical research in the modern healthcare context [53, 54-55]. A historical normative analysis by Adashi *et al.* (2018) tracing the evolution of the report over four decades and its lasting impact on ethical principles in clinical research indicates that the commission was created in 1974 following the public outrage over the Tuskegee Syphilis Study, which had a detrimental effect on study subjects due to the unethical approach used [56]. The Belmont Report contributed to the advancement of ethics regulations in research by introducing respect for persons, justice and beneficence as key principles in clinical and behavioral research. The report also guides subject selection and evaluation of study risks. The report remains the primary code underlying ethical research in the US.

## The limitations of informed consent in clinical research:

Most people who consent to clinical trials lack the adequate decisional capacity to consent to research [57, 58-59]. Decisional capacity refers to a potential participant's ability to logically process information pertaining to IC and consent to participation in a study from a point of knowledge [60-61]. A systematic review of empirical studies on the effectiveness of IC in clinical research by Pietrzykowski and Smilowska (2021) found that human subject's level of understanding of IC's basic components is limited [2]. The researchers concluded that the study findings should worry researchers, since a lack of comprehension undermines the ethical pillar of contemporary clinical trial practice [2]. Similarly, a cross-sectional study by Agozzinno *et al.* (2019) explored whether the ICF adequately informs surgical patients before they sign and found that close to half of the patients consented to theater procedures without intensively reading the IC document to understand [62]. Pietrzykowski and Smilowska (2021) found that the issue of physicians overrating their patients' level of understanding is common and in some instances, the patients overestimate their own level of comprehension, leading to their enrolment in trials that they hardly comprehend [2]. Poor participants' understanding of the IC component casts doubt on their complete and genuine involvement in the shared medical decision-making process. Therefore, relying on IC is insufficient because some participants are unable to comprehend the fundamental IC components.

The lack of agreement on what entails adequate IC makes it possible for researchers to manipulate/trick patients into agreeing to participate in studies without properly comprehending the associated risks in the trials [63, 64-65]. Wisgalla and Hasford (2022) critically analyzed current clinical research practices to present reasons why most ICs in clinical research are invalid and found that standard measures of how the ICF should be written are lacking [66]. Wisgalla and Hasford (2022) critically analyzed current clinical research practices to present reasons why most ICs in clinical research are invalid and found that standard measures of how the ICF should be written were lacking [66]. The authors report that such loopholes are exploited by researchers who craft documents with low readability for clients, leading to the signing of consent forms without a comprehensive understanding of the risks and benefits of participating in the trials. Wisgalla and Hasford (2022) revealed that some practitioners provide patients with IC documents that are too long to be read completely and others have sophisticated language or poor handwriting; thus, clients fail to identify the material facts concerning a trial [66]. The findings concur with an earlier observational cross-sectional study by Schumacher *et al.* (2017), which investigated whether patients with cancer effectively comprehend the critical components of the ICF before enrolling in clinical trials [67]. Many participants enrolled in cancer trials have poor comprehension of the essential elements of their trial and their situation worsens when poorly formulated ICFs with low readability are provided [67]. The lack of standardization regarding how to design and present the ICF creates a loophole for unscrupulous researchers to enroll participants who barely understand the nature and risks of the clinical research in which they participate.

The main limitation of the findings of Pietrzykowski and Smilowska (2021) is that most of the studies selected for the review used small sample sizes, thus reducing their statistical power [2]. Furthermore, the studies were generally based on ethnically and socially heterogeneous samples of patients with similar types of underlying medical issues [68-69]. Therefore, it is plausible to conclude that patients' pre-existing medical conditions may affect their comprehension of the IC [70]. Schumacher *et al.* (2017) cited potential selection bias that could have affected the overall distribution of variables since most participants were homogenous in terms of race/ethnicity and native language and most respondents were enrolled in the study many weeks after giving consent to their treatment trial, heightening the risk of recall bias [67]. Agozzino *et al.* (2019) cited potential recall bias among participants since information was obtained from them through face-to-face interviews multiple days after signing the IC and being discharged, leading to a situation where some people could not remember clearly if they understood the document [62]. Furthermore, the statistical power across all studies was limited by the sample size. Due to these limitations, the study findings can be challenged as being only hypothesis-generating rather than definitive. Consequently, future studies examining patients' consent capacity should minimize the time between signing the consent form and participation in a study and be intentional about strategies to overcome selection biases to produce definitive and generalizable results.

## The need for a comprehensive ethical framework:

A new and comprehensive ethical framework is necessary to ensure that the ethical approaches adopted by clinical researchers address the challenges presented by modern technology and complex medical situations [71, 72, 73-74]. Vayena *et al.* (2023) and Van Rijssel *et al.* (2024) observed that the current ethical framework on Decentralised clinical trials (DCT) focuses majorly on the four elements of the IC procedure that are affected by DCTs, which makes it inefficient for complex technology-enhanced clinical trials [75]. Consequently, the researchers argue for the need to create a more comprehensive ethical framework with a broader view of the ethical aspects of DCTs to address the new challenges presented by the growth of DCT practice [75-76]. Similarly, a narrative review by van Bruchem-Visser *et al.* (2020) analyzed 17 different ethical frameworks and concluded that extant ethical guidelines for medical professionals and researchers are often limited and unable to address ethical issues, especially when complex medical decisions should be made [77]. Consequently, van Bruchem-Visser *et al.* (2020) suggested the need for more advanced ethical frameworks that can address the challenges that emerge during complex medical decision-making, especially when dealing with older patients [77]. A study by Xu *et al.* (2020) investigated Australian researchers' experiences and views on obtaining IC and concluded that there is a need for a more comprehensive ethical framework that can provide more updated ethical support to researchers [78]. Xu *et al.* (2020) asserted that improvements in existing guidelines by Human Research Ethics Committees (HRECs) are necessary to guide researchers in designing IC procedures and lay a blueprint for improved communication between researchers and HRECs [78]. Owing to the limitations of IC, which is mistakenly regarded as the cornerstone of ethical conduct and regulation of research, there is a need for a systematic and consistent framework that integrates all pertinent ethical factors in clinical research.

## Systematic framework for responsible clinical research:

Deriving from the basic philosophies underlying major codes, declarations and guidelines relevant to research on human subjects, there is need to adopt a consistent ethical framework that ensures ethical principles are combined with eight prerequisites for ethical clinical research underpinned by conventional ethical principles. The idea for the eight requirements may be drawn from the recommendations of researchers such as Sutrop and Lõuk (2020) [79] and Emanuel *et al.* (2000) [32], who challenge the validity of current ethical guidelines. The researchers contend that a comprehensive ethical framework should be based on the following eight prerequisites: collaborative partnership, favorable risk/benefit ratio, scientific/social value, fair subject selection, scientific validity and independent review, respect for participants and communities and informed consent [32, 79]. Sutrop and Lõuk (2020) emphasized that, in addition to principles guiding research on clinical trials, an ethical framework for research should provide more than principles so that it can be effective in confronting unanswered questions [79]. The benchmarks are drawn from studies by researchers such as van Rijssel *et al.* (2024) [76] and van Bruchem-Visser *et al.* (2020) [77], who have continued to acknowledge the need for a more broadened and updated ethical framework that can guide the development of research designs and protocols intended to handle human subjects with care while respecting their rights and optimizing their safety and well-being. The eight prerequisites, as discussed in Table 1 (see PDF), form the basis for developing a comprehensive framework.

## Pertinent ethical principles and benchmarks for comprehensive ethical framework:

A comprehensive framework must constitute broad elements of moral values and guide researchers and practitioners about how to balance them while performing clinical research. A comprehensive framework must also ensure that conclusions can be made clear to all stakeholders and provide a procedural plan for structuring moral deliberation during research, especially in complex decision-making situations in clinical treatment whereby the use of technology is involved (such as in the case of DCTs).

## Conclusion:

While the IC is considered the cornerstone of clinical research, it does not, on its own, suffice to ensure that participants' rights, safety, and well-being are protected. There is a need to develop a comprehensive, systematic and consistent ethical framework beyond the IC that can integrate essential prerequisites underpinned by various ethical principles to enhance ethical compliance in clinical research.

## Authors' contribution:

R.W. and Y.A.K. contributed to the conceptualization of the study. R.W. wrote the original draft. Y.A.K. took part in writing review and editing. Both authors have read and approved the final version of the manuscript.

## Data availability:

No new data was created or analyzed, and therefore, data sharing is not applicable.
